# Association of *LIN28B* polymorphisms with chronic hepatitis B virus infection

**DOI:** 10.1186/s12985-020-01353-7

**Published:** 2020-06-22

**Authors:** Qunying Han, Jiao Sang, Xiude Fan, Xiaoyun Wang, Lu Zeng, Xiaoge Zhang, Kun Zhang, Na Li, Yi Lv, Zhengwen Liu

**Affiliations:** 1 Department of Infectious Diseases, First Affiliated Hospital of Xi’an Jiaotong University, Xi’an, 710061 Shaanxi People’s Republic of China; 2 Department of Hepatobiliary Surgery, First Affiliated Hospital of Xi’an Jiaotong University, Xi’an, Shaanxi People’s Republic of China; 3 Institute of Advanced Surgical Technology and Engineering, Xi’an Jiaotong University, No. 277 Yanta West Road, Xi’an, 710061 Shaanxi Province People’s Republic of China

**Keywords:** Chronic hepatitis B virus infection, Hepatocellular carcinoma, Gene polymorphisms, *LIN28B*

## Abstract

**Background:**

LIN28B is involved in multiple cellular developmental processes, tissue inflammatory response and tumourigenesis. The association of *LIN28B* polymorphisms with hepatitis B virus (HBV) infection remains unknown.

**Methods:**

This study investigated the association of *LIN28B* rs314277, rs314280, rs369065 and rs7759938 polymorphisms in patients with chronic HBV infection, a major cause of liver disease including hepatocellular carcinoma (HCC). A total of 781 individuals including 515 cases of chronic HBV infection (91 asymptomatic carrier status, 128 chronic hepatitis, 127 cirrhosis and 169 HCC), 97 HBV infection resolvers and 169 healthy controls were investigated.

**Results:**

*LIN28* rs314280 genotypes GA + AA were higher in resolver and controls than patients (*P =* 0.011). Patients had significantly lower rs314280 allele A than resolvers (*P =* 0.031, OR 0.689, 95%CI 0.491–0.969) or controls (*P =* 0.034, OR 0.741, 95%CI 0.561–0.978). In dominant model, patients had significantly lower rs314280 genotypes AA+GA than controls (*P =* 0.008, OR 0.623, 95%CI 0.439–0.884). *LIN28* rs7759938 genotypes TC + CC were higher in resolvers and controls than patients (*P =* 0.015). Patients had significantly lower rs7759938 allele C than resolvers (*P =* 0.048, OR 0.708, 95%CI 0.503–0.999). In dominant model, patients had significantly lower rs7759938 genotypes TC + CC than controls (*P =* 0.010, OR 0.632, 95%CI 0.445–0.897). Chronic hepatitis patients had lower frequency of rs369065 genotype TC than asymptomatic carriers, cirrhosis and HCC (*P =* 0.019).

**Conclusions:**

These results suggest that *LIN28* rs314280 and rs7759938 may be related to the susceptibility of chronic HBV infection. Further studies are warranted to examine the association of *LIN28B* polymorphisms with HBV-related diseases, especially HCC.

## Background

RNA-binding protein lin28 was initially identified as a regulator of developmental timing in *Caenorhabditis elegans* [1]. Subsequent studies revealed that in mammalian embryonic stem cells, LIN28 proteins regulate the self-renewal through suppression of *let-7* microRNAs (miRNAs) and influence of mRNA translation. LIN28 proteins also play important roles in many other biological processes [2–4]. Studies have indicated that LIN28 links inflammation to cell transformation and cancer [5, 6]. There are two homologous LIN28 proteins in mammals: LIN28A and LIN28B, that play crucial roles in diverse cellular developmental processes, tissue inflammatory response and tumorigenesis [5, 7–9]. Different from LIN28A, LIN28B acts solely in the nucleus by sequestrating primary *let-7* transcripts and suppressing their processing [9]. LIN28B is more wide-rangingly expressed in various tissues and highly expressed in placenta, fetal liver and testis [10]. LIN28B may be overexpressed in some tumors such as colon cancer [9, 11]. LIN28B enhances cell migration, invasion and metastasis in some cancers such as prostate cancer [12] and colon cancer [13]. LIN28B is associated with aggressive subtypes in certain cancers including high-grade serous ovarian cancers [14], esophageal cancer [15] and colon cancer [9, 13]. LIN28B overexpression is related to reduced patient survival and increased probability of tumor recurrence in colon cancers [16].

In terms of hepatitis B virus (HBV), a major cause of liver disease including hepatocellular carcinoma (HCC) worldwide, LIN28B is involved in the regulation of HBV replication and plays an oncogenic role in HCC. Studies have demonstrated that LIN28B is involved in miRNA-125b-5p mediated post-transcriptional regulation of HBV replication [17]. LIN28B over-expression mediates the suppression of let-7 expression by HBV X protein (HBx) in HepG2 hepatoma cells [18–20]. *LIN28B* serves as a key driver gene in HBx-induced hepatocarcinogenesis [21]. LIN28B is overexpressed in HCC [10, 18, 22] and its expression promotes transformation and invasion of HCC [18] while the suppression of *LIN28B* expression inhibits HCC cell proliferation and metastasis [23]. Higher expression of LIN28B is associated with shorter overall survival in HCC patients [22]. LIN28B in the peripheral blood mononuclear cells is shown to be an oncofetal circulating cancer stem cell-like marker associated with recurrence of HCC [24].

The relationship of *LIN28B* polymorphisms to cancer development, progression and metastasis, and patient prognosis, has also been investigated in some cancers. For example, the *LIN28B* rs314277 polymorphism was shown to have a significant association with risk of recurrence in patients with stage II colorectal cancer [25]. Although LIN28B has been demonstrated to be involved in HBV replication [17], HBV–related hepatocarcinogenesis [18–21] and the migration, invasion, metastasis and prognosis in HCC [18, 22, 24], the association of *LIN28B* polymorphisms with the susceptibility of chronic HBV infection and the clinical disease of chronic HBV infection including the development of HCC remains unknown. Therefore, this study investigated the relationship between *LIN28B* polymorphisms and the susceptibility of chronic HBV infection and HBV-related liver disease including HCC in Han Chinese population.

## Methods

### Study populations

In total, 515 patients with chronic HBV infection, 97 individuals with spontaneously resolved HBV infection and 169 healthy control individuals from the First Affiliated Hospital of Xi’an Jiaotong University were included in this study. Patients with chronic HBV infection were those who had HBsAg positivity for more than 6 months. Patients infected with other hepatitis viruses (hepatitis A, C, D and E), patients with severe heart, lung, brain, kidney and other important organ diseases which were not related to HBV infection, patients with other liver disease such as alcoholic liver disease, non-alcoholic fatty liver disease, drug-induced liver injury and autoimmune liver disease and patients under 18 years of age were excluded. HBV infection resolvers were individuals who spontaneously resolved from HBV infection with normal liver function, HBsAb and HBcAb positivity, and undetectable HBV DNA. The healthy control individuals were healthy blood donors or those who had normal results of regular physical examination. All subjects were unrelated Han Chinese. All subjects signed informed consent and volunteered to participate in the study. The study was performed in accordance with the Declaration of Helsinki and the study was approved by the Ethics Committee of the First Affiliated Hospital of Xi’an Jiaotong University.

### Genotyping of the polymorphisms

In the dbSNP database and Hapmap database of NCBI, *LIN28B* SNPs with minor allele frequency > 0.03 and r^2^ > 0.8 (rs314277, rs314280, rs369065, and rs7759938) were selected for genotyping. Venous blood was collected from all subjects in the morning after overnight fasting. About 2 ml of whole blood was collected in a tube containing EDTA anticoagulant and stored below − 30 °C.

Genomic DNA was extracted using TIANGEN blood genomic DNA extraction kit following the instructions of the manufacturer (Beijing Tiangen Biochemical Technology Co., Ltd., Beijing, China). The genotyping of the polymorphisms was performed using genomic DNA by the propriety multiplex SNP genotyping system SNPscan (Genesky Biotechnologies Inc., Shanghai, China). This system was developed according to an SNP genotyping technique based on double ligation and multiplex fluorescence polymerase chain reaction (PCR) [26]. The PCR products were analyzed using GeneMapper 4.1 software (Applied Biosystems, USA). The sequences of the probes and primers used for rs314277, rs314280, rs369065, and rs7759938 genotyping are listed in Table S1. Based on the results, rs314277 was genotyped as genotypes CC, CA and AA, rs314280 as genotypes GG, GA and AA, rs369065 as genotypes TT, TC and CC, and rs7759938 as genotypes TT, TC and CC.

### Statistical analysis

Statistical analysis was performed using SPSS 18.0 software (SPSS Inc. Chicago). The t-test or χ^2^ test was used to compare the clinical and laboratory data between groups where appropriate. The common homozygote was used as reference. Odds ratio (OR) with 95% confidence interval (CI) was calculated using univariate logistic regression between genotype and allele frequencies of different groups under different genetic models. Haplotypes were estimated by the SHEsis method (http://Analysis.Bio-x.Cn). *P* values < 0.05 in multiple comparisons were corrected by Bonferroni method and presented as corrected *P* value (*P*c). *P* values or *P*c values < 0.05 were considered statistically significant.

## Results

### Charecteristics of the study population and hardy-Weinberg equilibrium

The gender between patients with chronic HBV infection (male/female, 360/155), HBV infection resolvers (male/female, 65/32) and healthy controls (male/female, 105/64) had no significant difference (*P =* 0.168). The age between patients with chronic HBV infection [41.87 ± 13.30 (18–78) years], HBV infection resolvers [40.04 ± 13.22 (28–72) years] and healthy controls [43.38 ± 13.25 (24–76) years] also had no significant difference (*P =* 0.136, Table 1). The clinical diagnosis of the 515 patients with chronic HBV infection included 91 asymptomatic HBV carrier status, 128 chronic hepatitis, 127 cirrhosis and 169 HCC (Table 1).

**Table 1:** Gender and age in patients with chronic HBV infection, HBV infection resolvers and healthy controls and the clinical diagnosis of the patients with HBV infection

	Patients (*n* = 515)	Resolvers (*n* = 97)	Controls (*n* = 169)	*P*
Gender (male/female)	360/155	65/32	105/64	0.168
Age [mean (range) years]	41.87 ± 13.30 (18–78)	40.04 ± 13.22 (28–72)	43.38 ± 13.25 (24–76)	0.136
Clinical diagnosis				
Asymptomatic carrier status	91			
Chronic hepatitis	128			
Cirrhosis	127			
Hepatocellular carcinoma	169			

Patients, patients with chronic HBV infection; resolvers, HBV infection resolvers; controls, Healthy controls. The t-test or Chi-squared test was used for comparison

The polymorphisms (rs314277, rs314280, rs369065, and rs7759938) in the study participants were all successfully genotyped. The genotype frequencies of the patients with chronic HBV infection, HBV infection resolvers and healthy controls were in accordance with the Hardy-Weinberg equilibrium (P > 0.05, Table S2).

### Genotype and allele frequencies of the polymorphisms in patients with chronic HBV infection, HBV infection resolvers and healthy controls

The frequencies of rs314277 genotype CC, CA and AA in patients with chronic HBV infection, infection resolvers and healthy controls had no significant difference (Table S3). The frequencies of rs314280 genotype GG, GA and AA between patients with chronic HBV infection, infection resolvers and healthy controls were significantly different. Using genotype GG as reference, genotype GA frequency was higher in resolver and healthy controls than in patients (*P =* 0.012, Table S3). The rs314280 genotypes GA + AA were also higher in resolver and healthy controls than in patients (*P =* 0.011, Table S3). The frequencies of rs369065 genotype TT, TC and CC in patients with chronic HBV infection, infection resolvers and healthy controls had no significant difference (Table S3). The frequencies of rs7759938 genotype TT, TC and CC between patients with chronic HBV infection, infection resolvers and healthy controls were significantly different. Using genotype TT as reference, genotype TC frequency was higher in resolver and healthy controls than in patients (*P =* 0.011, Table S3). The rs7759938 genotypes TC + CC were also higher in resolver and healthy controls than in patients (*P =* 0.015, Table S3).

The frequency of rs314277 allele had no significant difference between patients with chronic HBV infection, HBV infection resolvers and healthy controls (*P =* 0.665, Table S4). The frequency of rs314280 allele A was higher in resolvers and healthy controls than in patients (*P =* 0.024, Table S4). The frequency of rs369065 allele had no significant difference between patients with chronic HBV infection, HBV infection resolvers and healthy controls (*P =* 0.356, Table S4). The frequency of rs7759938 allele C was higher in resolvers and healthy controls than in patients (*P =* 0.043, Table S4).

### Genotype and allele frequencies between patients with chronic HBV infection, HBV infection resolvers and healthy controls in different genetic models

The genotype and allele frequencies of rs314277 and rs369065 between patients with chronic HBV infection, HBV infection resolvers and healthy controls had no significant difference in any genetic models (Table S5, Table S6).

For genotype and allele frequencies of rs314280, patients with chronic HBV infection had significantly lower allele A than resolvers (*P =* 0.031, OR 0.689, 95%CI 0.491–0.969) or controls (*P =* 0.034, OR 0.741, 95%CI 0.561–0.978, Table 2). In codominant and overdominant model, patients had significantly lower genotype GA than controls (*P =* 0.005, *P*c = 0.010, OR 0.593, 95%CI 0.413–0.853 and *P =* 0.005, OR 0.603, 95%CI 0.423–0.859, respectively). In dominant model, patients had significantly lower genotypes AA+ GA than controls (*P =* 0.008, OR 0.623, 95%CI 0.439–0.884, Table 2). In additive model, the genotype frequencies of rs314280 were significantly different between patients and resolvers (*P =* 0.038, OR 0.705, 95%CI 0.507–0.981) or controls (*P =* 0.036, OR 0.744, 95%CI 0.564–0.981, Table 2). No significant difference was observed between controls and resolver in rs314280 genotype and allele frequencies (Table 2).

**Table 2:** *LIN28B* rs314280 genotype and allele frequencies in patients with chronic HBV infection, HBV infection resolvers and healthy controls

		Patients (*n* = 515)	Resolvers (*n* = 97)	Controls (*n* = 169)	Patients vs. resolvers		Patients vs. controls		Resolvers vs. controls	
					*P*	OR (95%CI)	*P*	OR (95%CI)	*P*	OR (95%CI)
Genotype										
Codominant	GG	313 (60.8)	49 (50.5)	83 (49.1)	Reference		Reference		Reference	
	GA	170 (33.0)	38 (39.2)	76 (45.0)	0.130	0.700 (0.441–1.113)	0.005^**a**^	0.593 (0.413–0.853)	0.535	0.847 (0.501–1.433)
	AA	32 (6.2)	10 (10.3)	10 (5.9)	0.074	0.501 (0.232–1.083)	0.668	1.178 (0.401–1.797)	0.271	1.694 (0.658–4.358)
Dominant	GG	313 (60.8)	49 (50.5)	83 (49.1)	Reference		Reference		Reference	
	AA+GA	202 (39.2)	48 (49.5)	86 (50.9)	0.059	0.659 (0.426–1.018)	0.008	0.623 (0.439–0.884)	0.826	0.945 (0.574–1.558)
Recessive	GG + GA	483 (93.8)	87 (89.7)	159 (94.1)	Reference		Reference		Reference	
	AA	32 (6.2)	10 (10.3)	10 (5.9)	0.148	0.576 (0.273–1.215)	0.889	1.053 (0.506–2.191)	0.196	1.828 (0.732–4.562)
Overdominant	GG + AA	345 (67.0)	59 (60.8)	93 (55.0)	Reference		Reference		Reference	
	GA	170 (33.0)	38 (39.2)	76 (45.0)	0.240	0.765 (0.489–1.196)	0.005	0.603 (0.423–0.859)	0.358	0.788 (0.474–1.310)
Additive	–	–	–	–	0.038	0.705 (0.507–0.981)	0.036	0.744 (0.564–0.981)	0.709	0.927 (0.624–1.378)
Allele										
	G	796 (77.3)	136 (70.1)	242 (71.6)	Reference		Reference		Reference	
	A	234 (22.7)	58 (29.9)	96 (28.4)	0.031	0.689 (0.491–0.969)	0.034	0.741 (0.561–0.978)	0.714	1.075 (0.730–1.584)

Data are presented as n (%). ^a^*P*_c_ (*P* value by Bonferroni correction) = 0.010. Genotypic association tests between groups assuming codominant, dominant or log-additive genetic models were carried out by univariate logistic regression using SNPstats and odds ratios (OR) with 95% confidence interval (CI) were calculated. Multiple testing correction was performed by the Bonferroni method

For genotype and allele frequencies of rs7759938, patients with chronic HBV infection had significantly lower allele C than resolvers (*P =* 0.048, OR 0.708, 95%CI 0.503–0.999, Table 3). In codominant and overdominant model, patients had significantly lower genotype TC than controls (*P =* 0.005, *P*_c_ = 0.01, OR 0.593, 95%CI 0.413–0.853 and *P =* 0.004, OR 0.594, 95%CI 0.418–0.850, respectively, Table 3). In dominant model, patients had significantly lower genotypes TC + CC than controls (*P =* 0.010, OR 0.632, 95%CI 0.445–0.897, Table 3). In additive model, the genotype frequencies of rs7759938 were significantly different between patients and controls (*P =* 0.033, OR 0.741, 95%CI 0.562–0.977, Table 3). No significant difference was observed between controls and resolver in rs7759938 genotype and allele frequencies (Table 3).

**Table 3:** *LIN28B* rs7759938 genotype and allele frequencies in patients with chronic HBV infection, HBV infection resolvers and healthy controls

		Patients (*n* = 515)	Resolvers (*n* = 97)	Controls (*n* = 169)	Patients vs. resolvers		Patients vs. controls		Resolvers vs. controls	
					*P*	OR (95%CI)	*P*	OR (95%CI)	*P*	OR (95%CI)
Genotype										
Codominant	TT	317 (61.6)	50 (51.5)	85 (50.3)	Reference		Reference		Reference	
	TC	166 (32.2)	38 (39.2)	75 (44.4)	0.113	0.689 (0.434–1.093)	0.005^**a**^	0.593 (0.413–0.853)	0.576	0.861 (0.510–1.454)
	CC	32 (6.2)	9 (9.3)	9 (5.3)	0.150	0.561 (0.253–1.245)	0.904	0.953 (0.438–2.074)	0.289	1.700 (0.633–4.565)
Dominant	TT	317 (61.6)	50 (51.5)	85 (50.3)	Reference		Reference		Reference	
	TC + CC	198 (38.4)	47 (48.5)	84 (49.7)	0.065	0.664 (0.430–1.028)	0.010	0.632 (0.445–0.897)	0.844	0.951 (0.577–1.567)
Recessive	TT + TC	483 (93.8)	88 (90.7)	160 (94.7)	Reference		Reference		Reference	
	CC	32 (6.2)	9 (9.3)	9 (5.3)	0.271	0.648 (0.299–1.404)	0.673	1.178 (0.550–2.521)	0.222	1.818 (0.696–4.748)
Overdominant	TT + CC	349 (67.8)	59 (60.8)	94 (55.6)	Reference		Reference		Reference	
	TC	166 (32.2)	38 (39.2)	75 (44.4)	0.184	0.739 (0.472–1.155)	0.004	0.594 (0.418–0.850)	0.409	0.807 (0.486–1.342)
Additive		–	–	–	0.058	0.725 (0.520–1.010)	0.033	0.741 (0.562–0.977)	0.848	0.962 (0.645–1.435)
Allele										
	T	800 (77.7)	138 (71.1)	245 (72.5)	Reference		Reference		Reference	
	C	230 (22.3)	56 (28.9)	93 (27.5)	0.048	0.708 (0.503–0.999)	0.051	0.757 (0.572–1.002)	0.738	1.069 (0.723–1.582)

Data are presented as n (%). ^a^*P*_c_ (*P* value by Bonferroni correction) = 0.010. Genotypic association tests between groups assuming codominant, dominant or log-additive genetic models were carried out by univariate logistic regression using SNPstats and odds ratios (OR) with 95% confidence interval (CI) were calculated. Multiple testing correction was performed by the Bonferroni method

### Haplotype frequencies in patients with chronic HBV infection, HBV infection resolvers and healthy controls

The four SNPs of the *LIN28B* gene constitute 12 haplotypes. The 4 haplotypes with a frequency greater than 0.03 were analyzed. The results show that the 4 haplotype frequencies between patients with chronic HBV infection, HBV infection resolvers and healthy controls had no significant difference but patients had a tendency of lower frequency of rs314277-rs314280-rs369065-rs7759938 haplotype C-A-C-C than resolver (*P =* 0.053) or controls (*P =* 0.069, Table S7).

### Associations between *LIN28B* polymorphisms and different clinical diseases

*LIN28B* rs369065 genotype TC frequency was significantly different between patients with different clinical disease, with chronic hepatitis patients having lower frequency than asymptomatic carriers, cirrhosis and HCC (*P =* 0.019, Table 4) but with no significant difference in allele frequencies. No significant differences were observed between the genotype and allele frequencies of rs314277, rs314280 and rs7759938 in patients with different clinical diseases (Table 4).

**Table 4:** Genotype and allele frequencies of *LIN28B* rs314277, rs314280, rs369065 and rs7759938 in patients with different clinical diseases

		AC (*n* = 91)	CH (*n* = 128)	LC (*n* = 127)	HCC (*n* = 169)	χ^2^	*P*
rs314277	Genotype						
	CC	84 (92.3)	124 (96.9)	117 (92.1)	159 (94.1)	Reference	
	AC	7 (7.7)	4 (3.1)	10 (7.9)	10 (5.9)	3.121	0.373
	AA	0 (0)	0 (0)	0 (0)	0 (0)	–	–
	Allele						
	C	175 (96.2)	252 (98.4)	244 (96.1)	328 (97.0)	Reference	
	A	7 (3.8)	4 (1.6)	10 (3.9)	10 (3.0)	3.024	0.388
rs314280	Genotype						
	GG	51 (56.0)	86 (67.2)	74 (58.3)	102 (60.4)	Reference	
	GA	34 (37.4)	33 (25.8)	45 (35.4)	58 (34.3)	4.203	0.240
	AA	6 (6.6)	9 (7.0)	8 (6.3)	9 (5.3)	0.313	0.958
	Allele						
	G	136 (74.7)	205 (80.1)	193 (76.0)	262 (77.5)	Reference	
	A	46 (25.3)	51 (19.9)	61 (24.0)	76 (22.5)	2.072	0.558
rs369065	Genotype						
	TT	40 (44.0)	70 (54.7)	50 (39.4)	79 (46.7)	Reference	
	TC	37 (40.7)	37 (28.9)	62 (48.8)	74 (43.8)	9.912	0.019
	CC	14 (15.4)	21 (16.4)	15 (11.8)	16 (9.5)	2.065	0.559
	Allele						
	T	117 (64.3)	177 (69.1)	162 (63.8)	232 (68.6)	Reference	
	C	65 (35.7)	79 (30.9)	92 (36.2)	106 (31.4)	2.711	0.438
rs7759938	Genotype						
	TT	53 (58.2)	88 (68.8)	72 (56.7)	104 (61.5)	Reference	
	TC	31 (37.1)	32 (25.0)	46 (36.2)	57 (33.7)	4.586	0.205
	CC	7 (7.7)	8 (6.3)	9 (7.1)	8 (4.7)	1.449	0.694
	Allele						
	T	137 (75.3)	208 (81.2)	190 (74.8)	265 (78.4)	Reference	
	C	45 (24.7)	48 (18.8)	64 (25.2)	73 (21.6)	3.802	0.284

*AC* chronic asymptomatic carrier, *CH* chronic hepatitis, *LC* liver cirrhosis, *HCC* hepatocellular carcinoma. Data are presented as n (%). Genotype frequencies were estimated using the direct counting method. Chi-square test was used for analysis

## Discussion

LIN28 not only plays key roles in multiple cellular developmental processes, but also is involved in tissue inflammatory response and tumourigenesis. In the present study, we evaluated the associations between *LIN28B* polymorphisms rs314277, rs314280, rs369065, and rs7759938 and chronic HBV infection in Chinese Han patients. We found that *LIN28B* rs314280 genotypes GA + AA and allele A and rs7759938 genotypes TC + CC and allele C were associated with a significantly decreased susceptibility of chronic HBV infection. In relation to clinical diseases, chronic hepatitis patients had lower frequency of rs369065 genotype TC than asymptomatic carriers, cirrhosis and HCC but no significant differences were observed in the genotype and allele frequencies of rs314277, rs314280 and rs7759938 between patients with different clinical diseases.

Rs314277 is an intronic polymorphism located in intron 2 of the *LIN28B* gene [27]. This polymorphism was shown to be associated with recurrence of colorectal cancer in Stage II disease [25]. However, in epithelial ovarian cancer, rs314277 was found to have no significant association with LIN28B levels or patient survival [28]. Correspondingly, we did not find any association between rs314277 polymorphism and the susceptibility of chronic HBV infection or the clinical diseases associated with HBV infection. It is suggested that the effect of rs314277 polymorphism may be disease-specific.

The rs314280 is located at the 5′ end of the *LIN28B* gene [27]. A previous study revealed that carriage of *LIN28B* rs314280 genotypes AG and AA/AG may increase the risk of grade ≥ 3 radiation-induced pneumonitis in patients with non-small cell lung cancer (NSCLC) [29]. We showed that *LIN28B* rs314280 genotypes GA + AA and allele A were associated with a significantly decreased susceptibility of chronic HBV infection. It is suggested that rs314280 polymorphism may have functional relevance and differentially affect the susceptibility to radiation-induced pneumonitis in NSCLC and chronic HBV infection.

*LIN28B* rs369065 was shown to be involved in the precocious puberty in Korean girls [30] although its function remains to be examined. The present study found that chronic hepatitis patients had lower frequency of rs369065 genotype TC than asymptomatic carriers, cirrhosis and HCC. This result implies the possibility that rs369065 may potentially influence liver inflammation in chronic HBV infection. However, the real association of rs369065 with chronic HBV infection and HBV-related disease requires additional studies considering the heterozygous nature of this genotype and the small sample size of populations in the subgroups of patients with different HBV-related diseases.

Previous studies showed that *LIN28B* rs7759938 was associated with neither breast cancer risk in European-American women [31] nor the severity of coronary lesions in a Chinese Han population [32]. Rs7759938 was indicated to have borderline significance in influencing LIN28B levels although it was not significantly associated with the survival of patients with epithelial ovarian cancer [28]. In the present study, we found that rs7759938 was associated with susceptibility of chronic HBV infection but it was not associated with the clinical diseases including HBV-related HCC. It is suggested that rs7759938 may have potential predisposing effect on chronic HBV infection but not disease progression related to HBV infection including HCC development.

LIN28B was shown to be involved in the post-transcriptional regulation of HBV replication [17]. The present study showed that *LIN28B* rs314280 and rs7759938 polymorphisms were associated with the susceptibility of chronic HBV infection. These results may support the role of LIN28B in regulation of HBV replication and infection. Studies have showed that LIN28B plays a key role in regulating inflammation by influencing IL-6 expression [5]. This study found that chronic hepatitis patients had lower frequency of rs369065 genotype TC than asymptomatic carriers, cirrhosis and HCC. This finding may imply the role of LIN28B in the hepatic inflammation of chronic HBV infection. Furthermore, LIN28B plays important roles in regulating cell proliferation and apoptosis and *LIN28B* is considered to act as an oncogene [10]. LIN28B is involved in an epigenetic switch that links inflammation to cell transformation [5], HBx-induced hepatocarcinogenesis [18–21], HCC development and progression [10, 18, 22, 23] and the prognosis of HCC patients [22]. However, this study did not find any relationship between the polymorphisms genotyped and the development of HBV-related HCC. It should be noted that the sample size of HCC patients in this study is relatively small. This study only genotyped 4 polymorphisms of *LIN28B* in Han Chinese patients and the potential effect of other polymorphisms on HCC development can not be excluded. Therefore, additional studies are required to genotype more polymorphisms in larger sample sizes of patient populations to clarify the relationship between LIN28B polymorphisms and HBV-related diseases including HCC.

Notably, polymorphisms in *LIN28B* may influence human growth and development. For example, *LIN28B* rs314277 has sex-specific effects on growth [33] and significantly associates with age at menarche in Asians [34]. *LIN28B* rs314280 is significantly associated with age at menarche in Asians [34] and European women [27] and may contribute to idiopathic central precocious puberty susceptibility in Chinese girls [35]. *LIN28B* rs369065 is associated with precocious puberty in Korean girls [30]. *LIN28B* rs7759938 is considered a pubertal timing-associated marker associated with age at thelarche and age at menarche [36], pubertal growth [37], prepubertal growth in females and final height in males [33], and adult height in both sexes [38, 39]. The rs7759938 also associates with idiopathic central precocious puberty risk in Chinese girls [35] and earlier age at menarche in women with polycystic ovarian syndrome [40]. Interestingly, in males with chronic HBV infection, earlier-onset puberty is shown to be associated with earlier HBeAg seroconversion, higher serum alanine aminotransferase levels, and a greater HBV viral load reduction [41]. Therefore, the *LIN28B* polymorphisms may potentially tag the roles of *LIN28B* gene in influencing the disease course of chronic HBV infection. Particularly, *LIN28B* rs7759938 has been revealed to be associated with serum testosterone levels and the genotypes related to higher LIN28B expression also associate with lower serum testosterone levels in adult humans [42]. The contribution of LIN28B to the regulation of sex hormone pathways might relate to many of the phenotypes linked to the gene in the human studies [42]. In terms of HBV infection, pathways related to androgen signaling may influence the risk of HBV-related HCC among men [43] and higher levels of androgen signaling, exhibited by higher testosterone levels, may be associated with an increased risk of HBV-related HCC in men [43–45]. Although the present study did not display any association of the polymorphisms genotyped with the development of HBV-related HCC, the effect of LIN28B polymorphisms on sex hormone pathways and the link with HBV-related HCC are interesting issues to be elucidated.

This study has several limitations. First, the sample sizes of patient and control populations included in the study are small and this may lead to bias in the analysis. Second, the study only genetyped 4 polymorphisms with minor allele frequency > 0.03 and r^2^ > 0.8 in the dbSNP database and Hapmap database of NCBI and there are other polymorphisms which may be relevant remain ungenotyped and this may miss some potentially significant polymorphisms. Third, this study included only Chinese Han population and there may be ethnic deviations in the polymorphisms. Fourth, no functional investigation concerning the polymorphisms was carried out and this may compromise the confidence of the findings. Therefore, further studies are definitely required to extend the studies between *LIN28B* polymorphisms and HBV-related diseases, especially HCC.

## Conclusions

This study showed that *LIN28B* rs314280 and rs7759938 polymorphisms may be associated with the susceptibility of chronic HBV infection in Han Chinese individuals. Further functional researches and multiple-center studies with large-scale samples are warranted to confirm and extend our findings. In particular, additional studies are needed to investigate more polymorphisms in larger sample sizes of patient populations with different ethic origin to clarify the association between *LIN28B* polymorphisms and HBV-related diseases including HCC.

## Supplementary information

**Additional file 1 Table S1.** Sequences of the probes and primers used for *LIN28B* rs314277, rs314280, rs369065 and rs7759938 genotyping.

**Additional file 2 Table S2.** Hardy-Weinberg equilibrium of *LIN28B* rs314277, rs314280, rs369065 and rs7759938 genotypes.

**Additional file 3 Table S3.** Genotype frequencies of *LIN28B* rs314277, rs314280, rs369065 and rs7759938 in patients with chronic HBV infection, infection resolvers and healthy controls.

**Additional file 4 Table S4.** Allele frequencies of *LIN28B* rs314277, rs314280, rs369065 and rs7759938 in patients with chronic HBV infection, infection resolvers and healthy controls.

**Additional file 5 Table S5.***LIN28B* rs314277 genotype and allele frequencies in patients with chronic HBV infection, HBV infection resolvers and healthy controls.

**Additional file 6 Table S6.***LIN28B* rs369065 genotype and allele frequencies in patients with chronic HBV infection, HBV infection resolvers and healthy controls.

**Additional file 7 Table S7.** Haplotypes with a frequency greater than 0.03 in patients with chronic HBV infection, HBV infection resolvers and healthy controls.

## Data Availability

The data sets generated and analyzed during this study are available from the corresponding author on reasonable request.

## Abbreviations

HBV: Hepatitis B virus
HCC: Hepatocellular carcinoma
HBx: Hepatitis B virus X protein
miRNAs: microRNAs
NSCLC: Non-small cell lung cancer
