# Supplementary material for: Association of LIN28B polymorphisms with chronic hepatitis B virus infection
Source: Virol J. 2020 Jun 22;17:81. doi: 10.1186/s12985-020-01353-7 (PMC7310063; doi:10.1186/s12985-020-01353-7)
Supplement: Supplementary file 1 — Additional file 1 Table S1. Sequences of the probes and primers used for LIN28B rs314277, rs314280, rs369065 and rs7759938 genotyping. [file 12985_2020_1353_MOESM1_ESM.doc]

Table S1. Sequences of the probes and primers used for *LIN28B* rs314277, rs314280, rs369065 and rs7759938 genotyping.

|  | Sequence | Length | Tm |
| --- | --- | --- | --- |
| rs314277 |  |  |  |
| Specific probe 1 | CF：CCATTTTCTCTCAGGCTCTTTCTTACC | 27 | 68.12 |
| Specific probe 2 | AF：CCATTTTCTCTCAGGCTCTTTCTTGCA | 27 | 67.71 |
| Universal primer | 3F：CCTTTGAACCTTCTTTAGGTTGTTAAATTG | 30 | 64.12 |
| rs314280 |  |  |  |
| Specific probe 1 | GR：CTTGCAGCAGAGGCACTTCAGAAGAC | 26 | 66.55 |
| Specific probe 2 | AR：CTTGCAGCAGAGGCACTTCAGAAGAT | 26 | 66.02 |
| Universal primer | 3R：AGTTGGGTCGCTTTGCTTTTG | 21 | 63.69 |
| rs369065 |  |  |  |
| Specific probe 1 | TR：A CCTG TR: ACCTGCTTTCTGATGGCATCGAA | 22 | 66.65 |
| Specific probe 2 | CR：CCTGCTTTCTGATGGCATCAAG | 22 | 66.34 |
| Universal primer | 3R：TCCAGAGCAAATCCCTGCTTC | 21 | 63.91 |
| rs7759938 |  |  |  |
| Specific probe 1 | TR：GCAGATGAAGCCTCAAAGGTAGGA | 25 | 66.83 |
| Specific probe 2 | CR：GCAGATGAAGCCTTCAAAGGTAGAAG | 25 | 66.56 |
| Universal primer | 3R：CCTTTAGAAAGAAAAAAATGGTAGTCTGTGG | 31 | 65.04 |
