# Supplementary material for: Association of LIN28B polymorphisms with chronic hepatitis B virus infection
Source: Virol J. 2020 Jun 22;17:81. doi: 10.1186/s12985-020-01353-7 (PMC7310063; doi:10.1186/s12985-020-01353-7)
Supplement: Supplementary file 2 — Additional file 2 Table S2. Hardy-Weinberg equilibrium of LIN28B rs314277, rs314280, rs369065 and rs7759938 genotypes. [file 12985_2020_1353_MOESM2_ESM.doc]

Table S2. Hardy-Weinberg equilibrium of *LIN28B* rs314277, rs314280, rs369065 and rs7759938 genotypes.

|  | Observed | | | Expected | | | χ^2^ | P |
| --- | --- | --- | --- | --- | --- | --- | --- | --- |
| rs314277 | AA | CA | CC | AA | CA | CC |  |  |
| Patients | 0 | 31 | 484 | 0 | 31 | 484 | 0.496 | 0.481 |
| Resolvers | 0 | 7 | 90 | 0 | 7 | 90 | 0.136 | 0.712 |
| Controls | 1 | 11 | 157 | 0 | 13 | 156 | 2.434 | 0.119 |
| rs314280 | AA | GA | GG | AA | GA | GG |  |  |
| Patients | 32 | 170 | 313 | 27 | 181 | 307 | 1.850 | 0.174 |
| Resolvers | 10 | 38 | 49 | 9 | 40 | 47 | 0.415 | 0.519 |
| Controls | 8 | 64 | 67 | 12 | 57 | 70 | 2.111 | 0.146 |
| rs369065 | CC | CT | TT | CC | CT | TT |  |  |
| Patients | 66 | 210 | 239 | 57 | 228 | 230 | 3.357 | 0.067 |
| Resolvers | 13 | 46 | 38 | 13 | 45 | 39 | 0.025 | 0.875 |
| Controls | 21 | 82 | 66 | 23 | 79 | 67 | 0.334 | 0.563 |
| rs7759938 | CC | CT | TT | CC | CT | TT |  |  |
| Patients | 32 | 166 | 317 | 26 | 179 | 310 | 2.579 | 0.108 |
| Resolvers | 9 | 38 | 50 | 8 | 38 | 50 | 0.206 | 0.650 |
| Controls | 9 | 75 | 85 | 13 | 67 | 89 | 2.142 | 0.143 |

Patients, patients with chronic HBV infection; resolvers, HBV infection resolvers; controls, healthy controls. Chi-square test was used for analysis.
