# Supplementary material for: Association of LIN28B polymorphisms with chronic hepatitis B virus infection
Source: Virol J. 2020 Jun 22;17:81. doi: 10.1186/s12985-020-01353-7 (PMC7310063; doi:10.1186/s12985-020-01353-7)
Supplement: Supplementary file 3 — Additional file 3 Table S3. Genotype frequencies of LIN28B rs314277, rs314280, rs369065 and rs7759938 in patients with chronic HBV infection, infection resolvers and healthy controls. [file 12985_2020_1353_MOESM3_ESM.doc]

Table S3. Genotype frequencies of *LIN28B* rs314277, rs314280, rs369065 and rs7759938 in patients with chronic HBV infection, infection resolvers and healthy controls.

|  | Patients  (n = 515) | Resolvers  (n = 97) | Controls  (n =169) | P |
| --- | --- | --- | --- | --- |
|
| rs314277 |  |  |  |  |
| CC | 484(94.0) | 90 (92.8) | 157 (92.9) | Reference |
| CA | 31(6.0) | 7 (7.2) | 11 (6.5) | 0.894 |
| AA | 0(0) | 0 (0) | 1 (0.6) | 0.162 |
| CA+AA | 31(6.0) | 7 (7.2) | 12 (7.1) | 0.831 |
| rs314280 |  |  |  |  |
| GG | 313 (60.8) | 49 (50.5) | 83 (49.1) | Reference |
| GA | 170 (33.0) | 38 (39.2) | 76 (45.0) | 0.012 |
| AA | 32 (6.2) | 10 (10.3) | 10 (5.9) | 0.204 |
| GA+AA | 202 (39.2) | 48 (49.5) | 86 (50.9) | 0.011 |
| rs369065 |  |  |  |  |
| TT | 239 (46.4) | 38 (39.2) | 66 (39.1) | Reference |
| TC | 210 (40.8) | 46 (47.4) | 82 (48.5) | 0.115 |
| CC | 66 (12.8) | 13 (13.4) | 21 (12.4) | 0.770 |
| TC+CC | 276 (53.6) | 59 (60.8) | 103 (60.9) | 0.149 |
| rs7759938 |  |  |  |  |
| TT | 317 (61.6) | 50 (51.5) | 85 (50.3) | Reference |
| TC | 166 (32.2) | 38 (39.2) | 75 (44.4) | 0.011 |
| CC | 32 (6.2) | 9 (9.3) | 9 (5.3) | 0.349 |
| TC+CC | 198 (38.4) | 47 (48.5) | 84 (49.7) | 0.015 |

Data are presented as n (%). Genotype frequencies were estimated using the direct counting method. Chi-square test was used for analysis.
