# Supplementary material for: Association of LIN28B polymorphisms with chronic hepatitis B virus infection
Source: Virol J. 2020 Jun 22;17:81. doi: 10.1186/s12985-020-01353-7 (PMC7310063; doi:10.1186/s12985-020-01353-7)
Supplement: Supplementary file 4 — Additional file 4 Table S4. Allele frequencies of LIN28B rs314277, rs314280, rs369065 and rs7759938 in patients with chronic HBV infection, infection resolvers and healthy controls. [file 12985_2020_1353_MOESM4_ESM.doc]

Table S4. Allele frequencies of *LIN28B* rs314277, rs314280, rs369065 and rs7759938 in patients with chronic HBV infection, infection resolvers and healthy controls.

|  | Patients  (n = 515) | Resolvers  (n = 97) | Controls  (n =169) | P |
| --- | --- | --- | --- | --- |
|
| rs314277 |  |  |  |  |
| C | 999(97.0) | 187 (96.4) | 325 (96.2) | Reference |
| A | 31(3.0) | 7 (3.6) | 13 (3.8) | 0.665 |
| rs314280 |  |  |  |  |
| G | 796 (77.3) | 136 (70.1) | 242 (71.6) | Reference |
| A | 234 (22.7) | 58 (29.9) | 96 (28.4) | 0.024 |
| rs369065 |  |  |  |  |
| T | 688 (66.8) | 122 (62.9) | 214 (63.3) | Reference |
| C | 342 (33.2) | 72 (37.1) | 124 (36.7) | 0.356 |
| rs7759938 |  |  |  |  |
| T | 800 (77.7) | 138 (71.1) | 245 (72.5) | Reference |
| C | 230 (22.3) | 56 (28.9) | 93 (27.5) | 0.043 |

Data are presented as n (%). Chi-square test was used for analysis.
