# Supplementary material for: Association of LIN28B polymorphisms with chronic hepatitis B virus infection
Source: Virol J. 2020 Jun 22;17:81. doi: 10.1186/s12985-020-01353-7 (PMC7310063; doi:10.1186/s12985-020-01353-7)
Supplement: Supplementary file 5 — Additional file 5 Table S5.LIN28B rs314277 genotype and allele frequencies in patients with chronic HBV infection, HBV infection resolvers and healthy controls. [file 12985_2020_1353_MOESM5_ESM.doc]

Table S5. *LIN28B* rs314277 genotype and allele frequencies in patients with chronic HBV infection, HBV infection resolvers and healthy controls.

|  |  | Patients  (n = 515) | Resolvers  (n = 97) | Controls  (n =169) | Patients *vs*. resolvers | | Patients *vs*. controls | | Resolvers *vs*. controls | |
| --- | --- | --- | --- | --- | --- | --- | --- | --- | --- | --- |
|  |  |  |  |  | P | OR (95%CI) | P | OR (95%CI) | P | OR (95%CI) |
| Genotype |  |  |  |  |  |  |  |  |  |  |
| Codominant | CC | 484 (94.0) | 90 (92.8) | 157 (92.9) | Reference |  | Reference |  | Reference |  |
|  | CA | 31 (6.0) | 7 (7.2) | 11 (6.5) | 0.654 | 0.823 (0.352-1.928) | 0.805 | 0.914 (0.449-1.861) | 0.835 | 1.110 (0.416-2.965) |
|  | AA | 0 (0) | 0 (0) | 1 (0.6) | - |  | - | - | - |  |
| Dominant | CC | 484 (94.0) | 90 (92.8) | 157 (92.9) | Reference |  | Reference |  | Reference |  |
|  | CA+AA | 31 (6.0) | 7 (7.2) | 12 (6.1) | 0.654 | 0.823 (0.352-1.928) | 0.615 | 0.838 (0.420-1.671) | 0.972 | 1.018 (0.387-2.678) |
| Recessive | CA+CC | 515 (100) | 97 (100) | 168 (99.4) | Reference |  | Reference |  | Reference |  |
|  | AA | 0 (0) | 0 (0) | 1 (0.6) | - |  | - | - | - |  |
| Overdominant | AA+CC | 484 (94.0) | 90 (92.8) | 158 (93.5) | Reference |  | Reference |  | Reference |  |
|  | CA | 31 (6.0) | 7 (7.2) | 11 (6.5) | 0.654 | 0.823 (0.352-1.928) | 0.818 | 0.920 (0.452-1.873) | 0.825 | 1.117 (0.418-2.984) |
| Additive |  | - | - | - | 0.837 | 0.912 (0.380-2.190) | 0.791 | 1.103 (0.534-2.280) | 0.703 | 0.830 (0.318-2.168) |
| Allele |  |  |  |  |  |  |  |  |  |  |
|  | C | 999 (97.0) | 187 (96.4) | 325 (96.2) | Reference |  | Reference |  | Reference |  |
|  | A | 31 (3.0) | 7 (3.6) | 13 (3.8) | 0.659 | 0.823 (0.352-1.928) | 0.449 | 0.776 (0.401-1.500) | 0.890 | 1.069 (0.419-2.725) |

Data are presented as n (%). Genotypic association tests between groups assuming codominant, dominant or log-additive genetic models were carried out by univariate logistic regression using SNPstats and odds ratios (OR) with 95% conﬁdence interval (CI) were calculated.
