# Supplementary material for: Association of LIN28B polymorphisms with chronic hepatitis B virus infection
Source: Virol J. 2020 Jun 22;17:81. doi: 10.1186/s12985-020-01353-7 (PMC7310063; doi:10.1186/s12985-020-01353-7)
Supplement: Supplementary file 6 — Additional file 6 Table S6.LIN28B rs369065 genotype and allele frequencies in patients with chronic HBV infection, HBV infection resolvers and healthy controls. [file 12985_2020_1353_MOESM6_ESM.doc]

Table S6. *LIN28B* rs369065 genotype and allele frequencies in patients with chronic HBV infection, HBV infection resolvers and healthy controls.

|  |  | Patients  [n (%)] | Resolvers  [n (%)] | Controls  [n (%)] | Patients *vs*. resolvers | | Patients *vs*. controls | | Resolvers *vs*. controls | |
| --- | --- | --- | --- | --- | --- | --- | --- | --- | --- | --- |
|  |  |  |  |  | P | OR (95%CI) | P | OR (95%CI) | P | OR (95%CI) |
| Genotype |  |  |  |  |  |  |  |  |  |  |
| Codominant | TT | 239 (46.4) | 38 (39.2) | 66 (39.1) | Reference |  | Reference |  | Reference |  |
|  | TC | 210 (40.8) | 46 (47.4) | 82 (48.5) | 0.178 | 0.726 (0.455-1.159) | 0.068 | 0.707 (0.487-1.027) | 0.925 | 0.974 (0.569-1.669) |
|  | CC | 66 (12.8) | 13 (13.4) | 21 (12.4) | 0.540 | 0.807 (0.406-1.603) | 0.621 | 0.868 (0.495-1.522) | 0.859 | 1.075 (0.484-2.389) |
| Dominant | TT | 239 (46.4) | 38 (39.2) | 66 (39.1) | Reference |  | Reference |  | Reference |  |
|  | TC+CC | 76 (53.6) | 59 (60.8) | 103 (60.9) | 0.189 | 0.744 (0.478-1.158) | 0.095 | 0.740 (0.519-1.055) | 0.984 | 0.995 (0.596-1.659) |
| Recessive | TT+ TC | 449 (87.2) | 84 (86.6) | 148 (87.6) | Reference |  | Reference |  | Reference |  |
|  | CC | 66 (12.8) | 13 (13.4) | 21 (12.4) | 0.874 | 0.950 (0.501-1.799) | 0.895 | 1.036 (0.613-1.751) | 0.819 | 1.091 (0.520-2.290) |
| Overdominant | CC+TT | 305 (59.2) | 51 (52.6) | 87 (51.5) | Reference |  | Reference |  | Reference |  |
|  | TC | 210 (40.8) | 46 (47.4) | 82 (48.5) | 0.224 | 0.763 (0.494-1.180) | 0.078 | 0.731 (0.515-1.036) | 0.863 | 0.957 (0.581-1.577) |
| Additive |  | - | - | - | 0.345 | 0.858 (0.624-1.179) | 0.240 | 0.859 (0.666-1.107) | 0.971 | 0.993 (0.681-1.449) |
| Allele |  |  |  |  |  |  |  |  |  |  |
|  | T | 688 (66.8) | 122 (62.9) | 214 (63.3) | Reference |  | Reference |  | Reference |  |
|  | C | 342 (33.2) | 72 (37.1) | 124 (36.7) | 0.291 | 0.842 (0.612-1.159) | 0.241 | 0.858 (0.664-1.109) | 0.922 | 1.019 (0.707-1.468) |

Patients, patients with chronic HBV infection; resolvers, HBV infection resolvers; controls, healthy controls. Data are presented as n (%). Genotypic association tests between groups assuming codominant, dominant or log-additive genetic models were carried out by univariate logistic regression using SNPstats and odds ratios (OR) with 95% conﬁdence interval (CI) were calculated.
