# Supplementary material for: Association of LIN28B polymorphisms with chronic hepatitis B virus infection
Source: Virol J. 2020 Jun 22;17:81. doi: 10.1186/s12985-020-01353-7 (PMC7310063; doi:10.1186/s12985-020-01353-7)
Supplement: Supplementary file 7 — Additional file 7 Table S7. Haplotypes with a frequency greater than 0.03 in patients with chronic HBV infection, HBV infection resolvers and healthy controls. [file 12985_2020_1353_MOESM7_ESM.doc]

Table S7. Haplotypes with a frequency greater than 0.03 in patients with chronic HBV infection, HBV infection resolvers and healthy controls.

| Haplotype  rs314277/ rs314280/  rs369065/ rs7759938 | Patients  (n = 515) | Resolvers  (n = 97) | Controls  (n =169) | P | Patients *vs.* Resolvers | | Patients *vs.* Controls | | Resolvers *vs.* Controls | |
| --- | --- | --- | --- | --- | --- | --- | --- | --- | --- | --- |
|  |  |  |  |  | P | OR (95%CI) | P | OR (95%CI) | P | OR (95%CI) |
| C-G-T-T | 677 (65.7) | 119 (61.3) | 211 (62.4) | Reference |  |  |  |  |  |  |
| A-A-C-C | 30 (2.9) | 6 (3.1) | 13 (3.8) | 0.624 | 0.788 | 1.138 (0.464-2.793) | 0.332 | 1.390 (0.712-2.714) | 0.692 | 1.222 (0.453-3.299) |
| C-A-C-C | 194 (18.8) | 49 (25.3) | 80 (23.7) | 0.057 | 0.053 | 1.437 (0.994-2.078) | 0.069 | 1.323 (0.977-1.791) | 0.701 | 0.921 (0.605-1.402) |
| C-G-C-T | 115 (11.2) | 17 (8.8) | 31 (9.2) | 0.699 | 0.533 | 0.841 (0.488-1.451) | 0.504 | 0.865 (0.565-1.324) | 0.931 | 1.028 (0.546-1.936) |

Data are presented as n (%). Haplotype construction was performed from the observed genotypes using SHEsis method (http://www.nhgg.org/analysis). Haplotype association tests between groups were carried out by univariate logistic regression and odds ratios (OR) with 95% conﬁdence interval (CI) were calculated.
